# Are biters sick? Health status of tail biters in comparison to control pigs

**DOI:** 10.1186/s40813-023-00314-0

**Published:** 2023-05-09

**Authors:** I. Czycholl, K. Büttner, D. Becker, C. Schwennen, W. Baumgärtner, W. Otten, M. Wendt, C. Puff, J. Krieter

**Affiliations:** 1 Institute of Animal Breeding and Husbandry, Kiel University, 24098 Kiel, Germany; 2 Pig Improvement Company (PIC), Hendersonville, TN 37075 USA; 3 Department for Animal Welfare and Disease Control, University of Copenhagen, 1870 Frederiksberg, Denmark; 4 Unit for Biomathematics and Data Processing, Faculty of Veterinary Medicine, Justus Liebig University, 35392 Giessen, Germany; 5 Institute of Genome Biology, Research Institute for Farm Animal Biology (FBN), 18196 Dummerstorf, Germany; 6 Clinic for Swine, Small Ruminants and Forensic Medicine and Ambulatory Service, University of Veterinary Medicine Hanover, Foundation, 30173 Hanover, Germany; 7 Department of Pathology, University of Veterinary Medicine Hanover, Foundation, 30559 Hanover, Germany; 8 Institute of Behavioural Physiology, Research Institute for Farm Animal Biology (FBN), 18196 Dummerstorf, Germany

**Keywords:** Abnormal behaviour, Behavioural disorder, Cannibalism, Health status, Pig, Tail biting

## Abstract

**Background:**

Tail biting is a multifactorial problem. As the health status is one of the factors commonly linked to tail biting, this study focuses on the health of identified biters. 30 (obsessive) biters are compared to 30 control animals by clinical and pathological examination as well as blood and cerebrospinal fluid samples. In that way, altogether 174 variables are compared between the groups. Moreover, connections between the variables are analysed.

**Results:**

In the clinical examination, 6 biters, but only 2 controls (P = 0.019) were noticeably agitated in the evaluation of general behaviour, while 8 controls were noticeably calmer (2 biters, P = 0.02). Biters had a lower body weight (P = 0.0007) and 13 biters had overlong bristles (4 controls, P = 0.008). In the pathological examination, 5 biters, but none of the controls had a hyperceratosis or inflammation of the pars proventricularis of the stomach (P = 0.018). However, 7 controls and only 3 biters were affected by gut inflammation (P = 0.03). In the blood sample, protein and albumin levels were below normal range for biters (protein: 51.6 g/l, albumin: 25.4 g/l), but not for controls (protein: 53.7 g/l, albumin: 27.4 g/l), (protein: P = 0.05, albumin: P = 0.02). Moreover, 14 biters, but only 8 controls had poikilocytosis (P = 0.05). Although not statistically different between groups, many animals (36/60) were affected by hypoproteinemia and hyponatremia as well as by hypokalemia (53/60) and almost all animals (58/60) had hypomagnesemia. For hypomagnesemia, significant connections with variables linked to tail damage and ear necrosis were detected (r_s_/V/ρ ≥ 0.4, P ≤ 0.05).

**Conclusion:**

The results suggest that behavioural tests might be helpful in identifying biters. Moreover, cornification and inflammation of the pars proventricularis is linked to becoming a biter. Furthermore, the results highlight the need for appropriate and adjusted nutrient and mineral supply, especially with regard to magnesium.

## Introduction

It is by now generally accepted that tail biting is a multifactorial problem [1, 2]. For example, access to feeder and drinker, food and water quality, feed composition, group composition, thermal comfort, handling, access to enrichment and rooting material, space, noise, genetics and the general health status as well as certain diseases have been linked to the occurrence of tail biting [3, 4]. While many studies have been carried out, up to now, no safe prevention or cure can be guaranteed. Instead, managing the respective risk factors on a farm-to-farm basis can probably be regarded as current state of the art [5]. Moreover, different hypotheses concerning the pathogenesis have been proposed [6]. However, given the multifactorial and multi-layered nature and complexity of the behaviour, it seems likely that also different forms of pathogenesis exist. In fact, Taylor et al. [3] proposed the existence of at least three different causative behaviours of tail biting: two-stage, sudden-forceful and obsessive. While the two-stage form can be linked to the high motivation to explore and a redirection of that motivation to pen mates, the sudden-forceful form is linked to resources and the inability to reach these resources (e.g. caused by feed outages). The obsessive form is specifically linked to single animals that become a biter. This form poses the major question why single animals of the same genetics, living under the same conditions as the others become tail biters. In fact, Taylor et al. [3] highlight the need for concentrating more on the biters in scientific studies. Indeed, in the last years, a significant rise in studies concentrating on the biter can be seen: For example, Zonderland et al. [7], Beattie et al. [8], Hoy et al. [9] and Brunberg et al. [10] have focussed on behavioural differences between biters and control animals. These studies registered for example more rope directed behaviour in a tail-chew test [8], a rise also in other abnormal behaviours such as belly nosing and manipulating other parts of the body of pen mates [9, 11]. In addition, Zonderland et al. [7] registered biters to be more often in a kneeling/sitting position. Some studies have developed and used comparable ethograms to identify biters [9, 11–13]. All this clearly demonstrates the possibility, but also the necessity to identify and focus more on the biters especially with focus on their health status. From other species, it is well known that the health status may well be linked to the development of abnormal behaviour patterns (e.g. [14–17]). Start for treatment of behavioural problems should therefore include a thorough clinical examination to reveal potential underlying medical causes [18, 19]. As already stated, health status is commonly named as risk factor in the occurrence of tail biting [20]. For example, Fritschen and Hogg [21] hypothesised that sickness-induced discomfort may be causative for the development of tail biting behaviour. Likewise, Moinard et al. [22] proved a correlation between diseases and tail biting occurrence on farms, which also led the authors to hypothesise that sickness-induced discomfort may cause tail biting. Therefore, in this study, we aimed at identifying biters and analysing their health status in comparison to animals not affected by tail biting. Therewith, we followed the hypothesis that subclinical diseases might cause the tail biter, in particular in the obsessive form of tail biting, to start this behaviour.

## Results

### Results of pairwise comparisons

In the time period of the study, on farm 1, three biters were identified, on farm 2, six and on farm 3, 21. Of all 60 animals, 26 were gilts (Biters: 15, Control: 11), 26 boars (Biters: 12, Control: 14) and 8 were castrates (Biters: 3, Control: 5). The weight of the animals ranged between 9.75 and 56 kg with a mean of 22.4 (biters) and 24.2 (controls) kg, respectively. Hence, most, but not all biters were identified in the rearing period. In Table 1, the mean prevalence (mean value incl. standard deviation for continuous variables and number of affected animals for binominal variables) is presented as well as the *P*-value resulting from the respective statistical analyses (depending on the distribution scale of each variable). It is a comparative presentation of the Biter and Control group. Significant differences were found for the following variables: More control animals [8] than biters (2) were scored as being specifically calm in the clinical observation (variable 2), while more biters [11] than controls (5) were scored as being specifically agitated (variable 3). Overlong bristles (variable 17) were found more often in biters [13] than controls [4]. More controls [5] than biters [1] had a partial tail loss (variable 27). In the clinical examination, on average, the biters were scored slightly lighter (BCS: 2.9 (± 0.4)) than controls (BCS: 3.1 (± 0.3)) (variable 33), which could be confirmed in the pathological examination, in which the body weight of the full carcass was assessed (variable 38) and on average, biters were lighter (22.4 (± 11.7) kg) than the controls (24.6 (± 12.0) kg). The adrenal gland weight (right: variable 40, left: variable 41) was significantly higher for the controls (right: 1.67 (± 0.66) g/left: 1.90 (± 0.91) vs. right: 1.45 (± 0.60) g/left: 1.63 (± 0.70)) and also when corrected for body weight, the relative adrenal gland weight of the right adrenal gland was still significantly higher in the controls (0.00007 (± 0.00001)) compared to the biters (0.000068 (± 0.00001)). While five biters, but no control animals were affected by a hyperceratosis of the pars proventricularis (variable 90), significantly more control animals [7] than biters [3] were affected by gut inflammation (variable 91). In the blood sample, although within normal range, biters had slightly lower values for mean corpuscular volume (MCHC, variable 120; 321.8 (± 58.9) g/l) than controls (335.0 (± 9.6) g/l), were more often affected by poikilocytosis (variable 132; 14 biters, 8 controls), had lower protein (variable 135; biters: 51.6 (± 4.8) g/l, controls: 53.7 (± 6.6) g/l) and albumin (variable 136; biters: 25.4 (± 5.2) g/l, controls: 53.7 (± 4.5) g/l) levels, higher creatinine kinase (CK, variable 138) levels (biters: 418.4 (± 1082.4) U/l, controls: 310.3 (± 190.0) U/l), slightly higher glucose (variable 147, biters: 6.1 (± 2.4) mmol/l, controls: 5.5 (± 1.9) mmol/l) and slightly lower phosphorus levels (variable 152; biters: 2.5, controls: 2.6). No significant differences were found in the analysis of the cerebrospinal fluid. Although no statistically significant differences between the groups were detected, it should further be noted that many animals (53/60; 25 biters, 28 controls) had a clinical anemia (variable 158), more than half (36/60; 20 biters, 16 controls) were affected by hypoproteinemia and hyponatremia, respectively and almost all animals (58/60; 29 biters, 29 controls) had a clinical hypomagnesemia (variable 160). Moreover, 53/60 animals had a hypokalemia (variable 162). It should further be noted that although the general aim was to identify control animals that were unaffected by tail biting, in the clinical examination five controls were affected by skin irritation on the tail, six controls by bleeding of the tail (variable 25) and five controls by partial tail losses (variable 27). Moreover, two controls were scored as having necrotic changes (variable 26) and eleven with crusts (variable 28) on the tail. Likewise, in the pathological examination, 18 controls were found with dermatitis (variable 94) and five with blood (variable 96) on the tail and another six were diagnosed to have tail necrosis (variable 97) and eleven were with crusts on the tail (variable 95). In all of these variables linked to the tail of the pigs, also biters were affected.

**Table 1:** Mean prevalence (including standard deviation or number of affected animals) of each variable as well as achieved *P*-value in the respective statistical comparisons for each variable as comparative presentation for the Biter and Control group

Code	Variable	Mean (± Std) / Prevalence (Biter)	Mean (± Std) / Prevalence (Control)	*P*-value
1	Rectal temperature	38.9 (± 0.4)	39.1 (± 0.4)	0.51
2	General behaviour: calm	2/30	8/30	**0.05**
3	General behaviour: agitated	11/30	5/30	**0.02**
4	Coughing	1/30	2/30	0.56
5	Sniffing	1/30	3/30	0.43
6	Nasal discharge	3/30	6/30	0.31
7	Snout	0/30	3/30	0.28
8	Ocular discharge	6/30	3/30	0.17
9	Ear veins	1/30	0/30	na
10	Respiratory rate	30.6 (± 8.9)	28.8 (± 9.9)	0.15
11	Heart rate	118.2 (± 23.1)	113.7 (22.7)	0.22
12	Heartbeat: intensity decreased	0/30	1/30	na
13	Heartbeat: intensity increased	1/30	1/30	1.00
14	Abdominal breathing	2/30	4/30	0.31
15	Feces	1/30	3/30	0.16
16	Skin condition	1/30	1/30	1.00
17	Bristles	13/30	4/30	**0.006**
18	Breathing noise (upper)	2/30	3/30	0.56
19	Breathing noise (medium)	14/30	12/30	0.47
20	Breathing noise (lower)	11/30	8/30	0.25
21	Conjunctivae: pale	6/30	10/30	0.06
22	Conjunctivae: reddened	3/30	1/30	0.15
23	Episcleral vessels	1/30	0/30	na
24	Tail: Skin irritation	2/30	5/30	0.17
25	Tail: Bleeding	4/30	6/30	0.47
26	Tail: Necrosis	3/30	2/30	0.65
27	Tail: Partial loss	1/30	5/30	**0.05**
28	Tail: Crust	11/30	11/30	1.00
29	Tail posture	10/30	11/30	0.70
30	Abdominal tension	3/30	4/30	0.70
31	Sunken flanks	1/30	1/30	1.00
32	Sheath	0/30	2/30	na
33	Body condition score	2.9 (± 0.4)	3.1 (± 0.3)	**0.05**
34	Lnn. inguinalis superficialis	15/30	16/30	0.25
35	Lameness	0/30	2/30	0.37
36	Joints	4/30	8/30	0.15
37	Hernia	0/30	2/30	0.44
38	Body weight	22.4 (11.7)	24.6 (12.0)	**0.0007**
39	Nutritional status: thin	3/30	1/30	0.09
40	Adrenal gland weight (right)	1.45 (0.6)	1.67 (0.66)	<** 0.0001**
41	Adrenal gland weight (left)	1.63 (0.7)	1.90 (0.91)	**0.002**
42	Relative adrenal gland weight (right)	0.000068 (0.01)	0.000070 (0.01)	**0.02**
43	Relative adrenal gland weight (left)	0.000078 (0.02)	0.000079 (0.02)	0.07
44	Skin: scratches	22/30	21/30	0.70
45	Skin condition	2/30	5/30	0.17
46	Ear: scratches	0/30	1/30	na
47	Umbilicus	0/30	2/30	na
48	Pneumonia	8/30	10/30	0.30
49	Lung: cyst	1/30	0/30	na
50	Lung: alveolar histiocytosis	9/30	8/30	0.56
51	Lung: hyperemia	12/30	14/30	0.65
52	Lung: edema	14/30	19/30	0.15
53	Lung: emphysema	4/30	6/30	0.31
54	Pleuritis	1/30	1/30	1.00
55	Peritoneal cavity: serous effusion	1/30	0/30	na
56	Liver: hyperemia	13/30	14/30	0.78
57	Liver: hematopoiesis	0/30	2/30	na
58	Liver: lymphohistiocytic inflammation	11/30	11/30	1.00
59	Myocarditis	4/30	5/30	0.73
60	Pericard: serous effusion	1/30	1/30	1.00
61	Diaphragma: lymphohistiocytic inflammation	2/30	1/30	0.71
62	Kidney: lymphohistiocytic inflammation	12/30	11/30	0.56
63	Kidney: cyst	2/30	1/30	0.56
64	Hydronephrosis	1/30	0/30	na
65	Kidney: hyperemia	3/30	2/30	0.56
66	Intraocular fluid	2/30	0/30	na
67	Bladder: lymphohistiocytic inflammation	1/30	1/30	na
68	Spleen: hyperplasia	6/30	3/30	0.17
69	Spleen: hyperemia	9/30	11/30	0.47
70	Bronchus associated lymphoid tissue: hyperplasia	9/30	7/30	0.25
71	Colon associated lymphoid tissue: hyperplasia	10/30	11/30	0.70
72	Colon associated lymphoid tissue: crypt abscesses	12/30	13/30	0.76
73	Colon associated lymphoid tissue: multinucleated giant cells	1/30	0/30	na
74	Pulmonal lymph nodes: hyperplasia	14/30	16/30	0.78
75	Pulmonal lymph nodes: purulent inflammation	1/30	1/30	1.00
76	Pulmonal hemosiderosis	1/30	1/30	na
77	Mesenteric lymph nodes: hyperplasia	23/30	22/30	0.47
78	Mesenteric lymph nodes: purulent inflammation	0/30	1/30	na
79	Tonsils: hyperplasia	19/30	20/30	0.65
80	Tonsils: crypt abscesses	23/30	23/30	1.00
81	Tonsils: purulent inflammation	0/30	1/30	na
82	Lnn. gastrici: sinus histiocytosis	0/30	1/30	na
83	Ln. sternalis: sinus histiocytosis	0/30	1/30	na
84	Tongue: inflammation	2/30	2/30	1.00
85	Tracheitis	7/30	8/30	0.76
86	Rhinitis	16/30	20/30	0.43
87	Nasal discharge	0/30	1/30	na
88	Atrophic conches	3/30	2/30	0.31
89	Gastritis	13/30	17/30	0.40
90	Pars proventricularis: hyperceratosis	5/30	0/30	**0.01**
91	Gut: inflammation	3/30	7/30	**0.03**
92	Gut: crypt abscesses	5/30	6/30	0.70
93	Gut: intraluminal inclusion corpuscles	1/30	3/30	0.15
94	Tail: dermatitis	15/30	18/30	0.43
95	Tail: crust	15/30	11/30	0.20
96	Tail: blood	3/30	5/30	0.31
97	Tail: necrosis	8/30	6/30	0.41
98	Tail: intralesional bacteria	4/30	3/30	0.65
99	Tail: exsudation	2/30	2/30	1.00
100	Tail: osteomyelitis	1/30	2/30	0.56
101	Ear necrosis	2/30	3/30	0.56
102	Skeletal musculature: inflammation	3/30	1/30	0.31
103	Joints: swelling/inflammation	1/30	1/30	1.00
104	Spinal cord: inflammation	2/30	1/30	0.56
105	Oesophagitis	3/30	3/30	1.00
106	Thymus: giant cell infiltration	1/30	0/30	na
107	N. ischiadicus: inflammation	0/30	1/30	na
108	Thyreoiditis	0/30	1/30	na
109	Sternal bone marrow: myeloid dominance	0/30	1/30	na
110	Plexus brachialis: internal bleeding	1/30	0/30	na
111	Pancreas: Hemorrhage	0/30	1/30	na
112	Cystitis	0/30	1/30	na
113	Prepuce: hyperkeratosis	1/30	0/30	na
114	Leukocytes [G/l]	18.2 (4.7)	19.3 (7.1)	0.36
115	Erythrocytes [T/l]	5.7 (0.6)	5.8 (0.5)	0.53
116	Hemoglobin [g/l]	98.2 (11.6)	100.3 (7.8)	0.31
117	Hematocrit [l/l]	0.30 (0.03)	0.29 (0.02)	0.51
118	Mean corpuscular volume (MCV) [fl]	51.7 (2.6)	51.3 (2.5)	0.65
119	Mean corpuscular hemoglobin (MCH) [pg]	17.2 (1.0)	17.2 (1.0)	0.69
120	Mean corpuscular hemoglobin concentration (MCHC) [g/l]	321.8 (58.9)	335.0 (9.6)	**0.05**
121	Thrombocytes [G/l]	523.9 (126.2)	513.6 (118.1)	0.60
122	Lymphocytes [%]	48.8 (14.6)	47.0 (13.0)	0.68
123	Segmented granulocytes [%]	45.6 (14.0)	47.9 (12.3)	0.44
124	Rod-nuclear granulocytes [%]	2.1 (2.1)	1.9 (2.0)	0.70
125	Metamyelocytes [%]	0.03 (0.12)	0.06 (0.21)	0.75
126	Eosinophilic granulocytes [%]	0.43 (0.71)	0.33 (0.40)	0.80
127	Basophilic granulocytes [%]	0.25 (0.34)	0.15 (0.26)	0.30
128	Monocytes [%]	2.63 (1.48)	2.55 (1.13)	0.86
129	Normoblasts [%]	0.23 (0.91)	0.16 (0.40)	0.67
130	Anisocytosis	22/30	26/30	0.17
131	Polychromacy	23/30	24/30	0.73
132	Poikilocytosis	14/30	8/30	**0.05**
133	Total bilirubin [µmol/l]	5.82 (4.24)	6.61 (4.77)	0.24
134	Conjugated bilirubin [mmol/l]	1.45 (2.12)	1.92 (2.05)	0.32
135	Protein [g/l]	51.6 (4.8)	53.7 (6.6)	**0.05**
136	Albumin [g/l]	25.4 (5.2)	27.4 (4.5)	**0.02**
137	Globulin: Albumin ratio	1.1 (0.4)	0.98 (0.28)	0.33
138	Creatinine kinase (CK) [U/l]	418.4 (1082.4)	310.3 (190.0)	**0.01**
139	Aspartate-amino-transferase (ASAT) [U/l]	16.1 (6.2)	17.9 (7.8)	0.34
140	CK: ASAT ratio	21.1 (28.6)	21.7 (19.6)	0.22
141	Glutamate dehydrogenase (GLDH) [U/l]	0.20 (0.82)	0.03 (0.11)	0.25
142	γ glutamyle transferase (GGT)	1.06 (4.1)	1.16 (4.4)	0.32
143	Alkaline phosphatase (AP) [U/l]	440.5 (115.6)	484.1 (126.6)	0.06
144	Creatinine [µmol/l]	86.1 (25.5)	79.5 (25.0)	0.16
145	Urea [mmol/l]	4.0 (1.6)	3.6 (1.4)	0.17
146	3-hydroxybutanoic acid (3HB)	0.10 (0.54)	0.006 (0.02)	0.32
147	Glucose [mmol/l]	6.14 (2.4)	5.5 (1.9)	**0.04**
148	L-lactate [mmol/l]	2.2 (0.9)	2.5 (1.0)	0.18
149	D-lactate [mmol/l]	0.02 (0.02)	0.01 (0.03)	0.28
150	Calcium (Ca) [mmol/l]	2.4 (0.24)	2.5 (0.2)	0.10
151	Magnesium (Mg) [mmol/l]	0.72 (0.08)	0.74 (0.08)	0.34
152	Phosphorus (P) [mmol/l]	2.5 (0.37)	2.6 (0.3)	**0.03**
153	Sodium (Na) [mmol/l]	137.8 (5.4)	138.1 (5.0)	0.66
154	Potassium (K) [mmol/l]	3.7 (0.46)	3.6 (0.4)	0.42
155	Ferric (Fe) [mmol/l]	15.8 (9.0)	16.0 (11.6)	0.91
156	Hemoglobin in plasma	0.19 (0.22)	0.52 (1.72)	0.49
157	Hemoglobin in serum	0.02 (0.1)	0.0 (0.0)	0.32
158	Anaemia	25/30	28/30	0.25
159	Hypoproteinemia	20/30	16/30	0.15
160	Hypomagnesemia	29/30	29/30	1.00
161	Hyponatremia	20/30	16/30	0.10
162	Hypokalemia	27/30	26/30	0.70
163	Hyperkalemia	1/30	0/30	na
164	Hypoglycemia	5/30	7/30	0.41
165	Hyperglycemia	11/30	8/30	0.17
166	Hypocalcemia	9/30	9/30	1.00
167	Leukopenia	1/30	1/30	na
168	Leucocytosis	5/30	8/30	0.25
169	Hyperphosphatemia	1/30	0/30	na
170	Dopamine [ng/ml]	1.2 (3.2)	1.4 (4.0)	0.36
171	3,4-Dihydroxyphenylacetic acid (DOPAC) [ng/ml]	3.0 (3.8)	2.8 (3.7)	0.59
172	5-Hydroxyindoleacetic acid (HIAA) [ng/ml]	19.6 (12.6)	24.2 (17.2)	0.30
173	Homovanillic acid (HVA) [ng/ml]	24.6 (28.1)	26.9 (25.2)	0.48
174	Serotonin	1.9 (7.4)	3.3 (8.5)	0.61

*na = statistics not calculated due to lack of variance

In some cases, statistics could not be calculated due to lack of variance (na). For an easier overview, significant *P*-values (≤ 0.05) are printed in bold

### Connection between collected variables

**Fig. 1:**
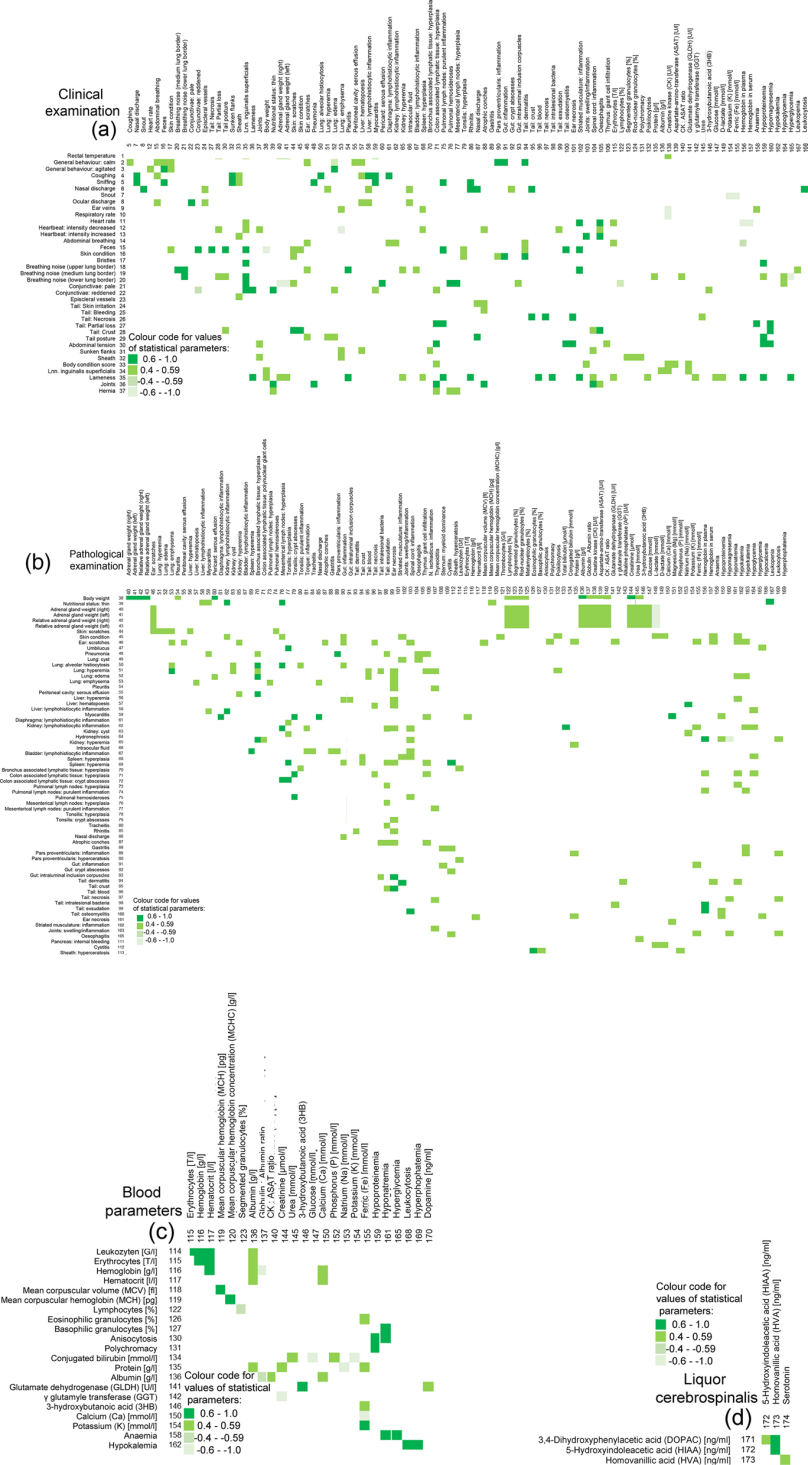
Connections between variables: clinical examination (**a**), pathological examination (**b**), blood sample (**c**), cerebrospinal fluid (**d**). Connections between all variables, calculated by Spearman’s correlation coefficient (numerical variables), Chi^2^ Test and Cramer’s V (binominal variables) and point-biserial correlation coefficient (connections between numerical and binominal variables). A connection was interpreted as meaningful if the values of the respective correlation coefficients were ≥ 0.4 (light green) and as strong if the values of the respective correlation coefficients were ≥ 0.6 (dark green) and *P*-values were ≤ 0.05. Negative connections are marked in transparent light and dark green colours, respectively. Due to the high number of variables, the figure is split in four parts according to the health variables under observation: **a** shows the variables of the clinical observation, **b** of the pathological examination, **c** of the blood tests and **d** of the analysis of the cerebrospinal fluid. Only those variables that had a connection to other variables are shown

Meaningful and strong connections between the variables are visualised in Fig. 1 a–d. Only for the variable 83 (Lnn. gastrici: sinus histiocytosis), 84 (Lnn. sternalis: sinus histiocytosis), 112 (Cystitis), 128 (Monocytes) and 129 (Normoblasts), no connections with other variables were found at all. Apart from that, most of the connections were positive and meaningful (respective statistical parameter: ≥ 0.4, colour code: light green), but not strong (respective statistical parameter: ≥ 0.6, colour code: dark green). Only few negative meaningful and strong connections were found.

## Discussion

Following the differentiation by Taylor et al. [3] into (1) two-stage (linked to the high exploration motivation of the pigs), (2) sudden-forceful (linked to stress induced by uncontrollable environment features such as sudden temperature in- or decreases, feed outages, water availability etc.) and (3) obsessive (single animals bite aimfully and forcefully into tails of pen mates without a visible reason), the aim of the present study was to focus and identify obsessive tail biters. As in general, the health status is commonly linked to tail biting outbreaks, the general hypothesis was that an impairment in the health status (potentially subclinical) is a causative factor for a specific animal to start tail biting, hence, in the identified obsessive tail biters, compared to the control animals, a deviation in the health status was expected. No further specification of that general hypothesis was possible, i.e. it was not focussed on a specific organ system, but a general overview of different health parameters was provided, which lead to a large amount of variables under observation (*n* = 174) linked to the general health status. Therefore, this study is to be seen as explorative study analysing the general link to the health status with the further aim to be able to highlight health parameters of specific importance to be analysed in more detail in future studies.

### Pairwise comparisons

In the general evaluation of behaviour during the clinical observation, significantly more biters were scored as specifically agitated, while significantly less biters were scored as specifically calm. Other studies have linked tail biting outbreaks with a generally higher level of activity and unrest a few days before a tail biting outbreak [23, 24]. Other authors have also found behavioural differences of tail biters [7–10]. These findings suggest that a standardised behavioural characterisation of tail biters, e.g. by behavioural tests for a better identification of tail biters may be possible in the future. Given the recent advances in precision livestock farming also in the pig industry [25], there is hope for a future automatic detection and early warning scheme for an animal to become a tail biter by behavioural characterisation. This is of specific importance, as the early identification and removal of biters from the group is currently probably the most effective management intervention strategy in tail biting [26]. Given the role of this study as a pilot study, the findings concerning the evaluation of general behaviour suggest that future studies should concentrate more on the behavioural differences, e.g. by proving secure identification of tail biters by standardised behavioural tests. This is further supported by the general knowledge that behaviour is one of earliest signs for changes [27, 28], the knowledge that capturing subtle behavioural changes is of utmost importance for early management intervention schemes [27] as well as the proven role of behaviour as iceberg indicator in welfare assessments in pigs [29, 30].

The weight of the adrenal glands was included as indicator, as there are hints from literature, that an enlargement can be seen as stress indicator [31, 32]. The expectation was that the tail biters were more prone to chronic stress and thus had larger adrenal glands. However, the opposite finding was made: the adrenal glands of the controls were significantly heavier. However, as also the body weight of the controls was higher, a correction for body weight was carried out. However, still, the right adrenal gland was significantly heavier in control animals. This may possibly be explained by an unsuitability of this indicator. Just looking at the size as single indicator may be a too simplified approach given the complexity of the biological endocrine systems. Probably, approaches such as the pathohistological examination with regard to the medulla:cortex ratio [33] or the gene expression in the adrenal glands [34, 35] would have been more suitable.

Significantly more biters were scored as having overlong bristles and in general, tail biters were significantly lighter than the control animals. This could be interpreted as tail biters being more often in the status of runts [36] and has already been hypothesized or reported by others [37, 38]. This may be a sign of a general shortage in nutritional requirements, such as nutrient or mineral supply or else, a health challenge in earlier stages of life. However, in the latter case, more anomalies in the pathological findings specifically of these animals would have been expected also when looking at the connections between the variables. So the more probable explanation is that for unknown reasons, nutritional requirements were not met. This comes as a surprise as all farms complied with national recommendations concerning feed composition in the different phases. However, it may be that lack of absorption occurred in these animals Kerr et al. [39] proved that pigs fed a lower protein diet showed an increase in activity level. Moreover, insufficient protein-content in feed and a generally increased activity level will lead to lesser weight gain (i.e. the animals will be the lighter ones) [40]. However, it should not stay unmentioned that despite the findings that biters were in general lighter than the controls, the general characterisation as an animal as being clinically thin (variable 39) failed to reach statistical significance in this study, which raises the question whether the small deviations in Body Condition Score and weight are really of biological significance. Moreover, although control pigs were randomly chosen that were of the same age class as the biters, this result may also be caused by the study design.

In general, one possible health challenge that might be linked to tail biting is a nutritional challenge, i.e. health issues of the gastrointestinal tract. This hypothesis is supported by the fact that tail biting in pigs often occurs around the time period two weeks after weaning [41]. Especially in pigs, weaning is carried out very abruptly and at a very early age which may well lead to severe nutritional adaptive challenges overstraining the adaptation capabilities and thus leading to behavioural disorders. However, while the finding that more biters are diagnosed with a hyperceratosis of the pars proventricularis supports this hypothesis, the fact that significantly more gut inflammations were observed in controls does not. Nevertheless, the pars proventricularis, the area of the stomach in which often gastric ulcers are observed in older pigs, should be observed in more detail also with regard to tail biting.

In the findings in the blood samples, the significant differences found for the mean corpuscular hemoglobin concentrations (variable 138; normal range: 300–350 g/l), glucose (variable 147; normal range: 4.0–6.4 mmol/l) and phosphorus (variable 153; normal range: 1.3–3.3 mmol/l) are most likely not of biological significance as they are so small and still well within the normal range in pigs. Although, the values for creatinine kinase (CK) (variable 138, normal range: 50–999 U/l) are well within the normal range and it is well known that already small influences by the sample collection [80], e.g. more flight attempts by the sample collection, may cause a rise, the difference is far more pronounced and especially the large standard deviation in biters in comparison to that of the control group should be noted. In contrast to that, more attention should be put on the protein (variable 135, normal range: 55–86 g/l) and albumin (g/l) levels in the blood sample, which were on average lower for the biters and below the reference levels (biters and controls for protein, only biters for albumin). The high need for meeting the nutritional requirements especially in fast growing breeds has already been highlighted by multiple authors [42] and moreover especially the protein and amino-acid requirements have been linked to tail biting outbreaks [4, 43]. However, clinical hypoproteinemia (variable 159), although present in more than half of all pigs (36/60) failed to reach significance in the comparison of the two groups. Hence, whether these findings are of biological significance and have explanatory power in the question, why obsessive tail biters start the behavioural disorder, must be confirmed in further studies. Another important finding from the blood samples was that significantly more biters were affected by poikilocytosis. Poikilocytosis describes an abnormality in erythrocyte forms. It has been linked to several diseases, specifically enteric diseases in pigs [44, 45]. Moreover, it has been linked to anemia caused by iron deficiency [46–48] and Konopel [49] has further linked it to a vitamin D deficiency. However, Christopher et al. [50] and Harvey [51] have suggested that poikilocytosis in young ruminants such as goats and cattle and maybe also pigs can be a normal finding. In the present study, the only connections of poikilocytosis was found to scratches on the ear (variable 46) and hypoproteinemia (variable 159). It should further be taken into consideration that in the present study, exactly half of affected pigs were scored as having mild poikilocytosis (≤ 33% of visible erythrocytes of abnormal shape) and the other half as moderate (≤ 66% of visible erythrocytes of abnormal shape) (results not shown), hence, the clinical relevance remains unclear.

In none of the findings, however, all biters were affected. Hence, the respective health issues cannot be the only explanation for an obsessive tail biter to be affected by the behavioural disorder.

The fact that many animals in this study (biters and controls alike) were diagnosed to have hypoproteinemia, hyponatremia and/or hypokalemia supports the hypothesis that tail biting may be linked to nutritional or mineral deficiencies. This holds despite the fact that national recommendations concerning feed composition in the different phases were followed carefully by all farms. Follow-up studies designed as controlled feeding-trials must clarify whether an adjustment of recommendations could be advisable and whether lack of absorption of nutrients could be the reason for these findings. Although no significant differences between the groups were detected, it should be born in mind that in the present study, all animals came from a population in which tail biting occurred. A special role therein plays magnesium, as almost all animals were diagnosed to have a clinical hypomagnesemia in this study. Some studies have proven beneficial effects of magnesium supplementation on different maladaptive behaviours in pigs [52–54]. However, another possible explanation for the high number of affected animals is that an adaptation of the reference values is needed, as usually, reference values are set from studies of limited pig population size and given ongoing advancements particularly in genetics and feeding may need regular adaptions.

### Connections between variables

Most relationships between variables were only moderate. Moreover, not all variables were well connected to each other (e.g. from the clinical and pathological examination). Hence, this analysis also proves that looking at this large variety of variables was (and is) necessary. Most connections were to be expected and can well be explained by already known linkages between health parameters. However, this detailed analysis also revealed some connections that seem to be of specific importance with regard to tail biting, which will be discussed in more detail in the following:

Changes in the tail and ear linked to necrotic findings are also linked to findings linked to respiratory diseases as well as abnormalities in the skin condition, whereby this holds especially for ear necrosis as well as tail dermatitis and blood on the tail. This linkage may well be explained by a generalised necrotic occurrence as for example already described in the 1980’s by Richardson [55], Schrauwen et al. [56] and Troxler [57] and named “Swine Inflammation and Necrosis Syndrome” recently by Reiner et al. [58]. However, Reiner et al. [58] described more signs of necrosis which were not observed in the present study, although Reiner et al. [58] also states that not always all signs are observed. However, this connection may also only be caused artificially without biological meaning, as these changes are changes of rather high occurrence and further signs for a generalised necrotic syndrome were not observed in the pathologic examination. While there is a connection between findings on the tail in the clinical and pathological examination, this does not hold for all tail related variables. This is most probably due to the fact that in the pathological examination, also pathohistological findings may be included, especially given necrotic findings. Another interesting observation is that the tail and ear associated variables (variables 25–30 and 96–103) are linked to anemia, hypoproteinemia, hypomagnesemia and hypokalemia (variables 159–163), underlining the already discussed importance of nutrition and mineral requirements with regard to tail biting.

### Limitations of the study

The first main limitation of this study is the small sample size. Altogether, 60 pigs were identified on three farms in Northern Germany. At the same time, on these animals, a large number of variables was assessed, which leads to the risk of α-error accumulation (for the pairwise comparisons as well as for the analysis of connections between the variables). However, given the pilot study character of this study, a larger sample size was not possible due to ethical considerations and moreover, as no information about the expected prevalences could be made beforehand. On the other hand, again given the pilot study character of this study, the large number of variables was necessary as no assumptions about expected organ systems could be made beforehand.

The second main limitation of this study is that all animals, biters as well as controls, were derived from farms in which, obviously, tail biting was present. This becomes also evident by tail damages of the control animals. Munsterhjelm et al. [59] also discussed this limitation. However, the problem is that tail biting is an unpredictable occurrence in pig husbandry. So basically, there is no farm that is 100% free from the risk of a tail biting outbreak in the future. At the same time, there is a need for studies for setting reference values and normal prevalences for the variables under study.

It must further be discussed whether there would have been even more variables of interest with regard to their connection to tail biting. For example, regarding the weight of the adrenal glands, potentially also a gene expression analysis or else a more thorough histological analysis of the cortex:medulla ratio might have yielded more insightful results. Likewise, analysing dopamine and serotonin content in the cerebrospinal fluid may be questioned as these neurotransmitters are not always freely measurable and furthermore may be dependent on the receptor density, 5-HT metabolism or gene expression in the brain [60–63].

## Conclusion

The aim of the present study was to answer the question whether a diminished health status was causative in the development of the behavioural disorder tail biting in pigs, i.e. the tail biters were affected by a – potentially subclinical – disease. No obvious affection of a specific organ system could be detected. The main findings include that biters differed significantly in their behaviour as compared to control animals, in particular their general behaviour was more often described as specifically agitated and significantly less often as calm. Moreover, although pairwisely allocated, i.e. of the same age class, biters were lighter than control pigs and had more often overlong bristles. This information gives hope that in the future, an easier identification of biters will become possible. It furthermore underlines the importance of understanding and watching out for subtle behavioural changes. Moreover, biters had more often a hyperceratosis of the pars proventricularis as well as a poikilocytosis, both findings need to be studied in more detail in the future as the link to tail biting remains unclear from the present results, especially as controversely, more controls had a gut inflammation. Although no significant differences between groups were found, many animals were in a nutritional and/or mineral deficiency, which highlights the link of tail biting to nutrition and underlines the importance of exact adaption of nutrition and mineral balance.

## Materials and methods

### Animals and variable collection

Data collection was carried out from May 2019 to February 2021 on three conventional farrow to finish pig farms in Schleswig–Holstein, Germany. All farms kept commercial cross breds (Duroc or Piétrain × (Large White × Landrace)). All animals were fed ad libitum, however, in dependency of the farm, the unit and the phase (rearing, growing, finishing) feeding differed (but it was the same for biters and respective associated control pigs). It was either mash or dry feed ad libitum with an animal to feeding place ratio of 1:1, 2:1 or 4:1. Two farms produced their own feed while one farm bought standard commercial feed from a local provider. Feed composition was in accordance with standard national recommendations (DLG, 2021) [64] for all phases and regularly checked by the farm managers as well as the respective advisory services. Two farms routinely castrated male pigs, the other farm raised intact boars and only castrated occasionally for educational purposes. Castration procedure was carried out according to German law requirements. All pigs that were identified for the study had undocked tails. One standard management intervention scheme in the case of tail biting on all farms was the early identification of biters and removal of those animals from the group. However, for standardisation, all involved staff members were trained to use a joint ethogram for identification of biters in this study. This ethogram worked in a two-stage process: in the affected pen, at least two different pigs with bleeding tails had to be present. These pens were then observed directly for 30 min. During this time frame, any tail-in-mouth behaviour was counted as tail biting event. To be identified as biter, one animal had to bite at least four times in the tails of at least two different pen mates. Upon identification of a biter, the animal was removed from the group. The biter as well as a control animal that was randomly chosen from a pen without known signs of tail biting (no damaging behaviour had been observed/noticed by the time) but of the same age class as the biter were then transported to the University of Veterinary Medicine, Hanover, Foundation, Germany (TiHo). After a resting period of about 2 h, the animals were examined by always the same person blind to the groups. Therefore, the standard protocol of the Clinic for Swine, Small Ruminants and Forensic Medicine and Ambulatory Service (TiHo) for veterinary clinical health checks was used. On the next day, the animals were put under ketamine-azaperone-injection anaesthesia (20 mg/kg bodyweight (BW) ketamine intra muscular (i.m.), Ketamin 100 mg/ml, CP-Pharma, Burgdorf, Germany, 2 mg/kg BW azaperone i.m., Stresnil 40 mg/ml, Elanco Germany GmbH, Bad Homburg, Germany) and blood samples were collected from the V. cava cranialis as well as cerebrospinal fluid samples lumbosacrally. Directly thereafter, the animals were euthanized by administering a letal dosis pentobarbital intravenously (80 mg/kg BW (< 30 kg BW) resp. 40 mg/kg BW (> 30 kg BW) pentobarbital intravenous (i.v., V. cava cranialis), Euthadorm 500 mg/ml, CP-Pharma, Burgdorf, Germany) and subjected to a thorough pathological (including histopathology) examination, which was carried out according to the standard protocol of the Department of Pathology (TiHo). Again, the examiners were blinded to the group (biter/control). The blood samples were further analysed by the standard in-house procedure of the laboratory of the Clinic for Swine, Small Ruminants and Forensic Medicine and Ambulatory Service of the TiHo. EDTA blood was used for a large blood count and Serum/Heparin plasma for a clinical-chemical analysis. Details on the in-house blood analysis can be found in Humann-Ziehank et al. [65]. Data concerning the blood values were evaluated using the internal species-specific reference values of the laboratory. Samples of the cerebrospinal fluid were stored at -73 °C and further processed later on. The storage period of the samples did not exceed six months. For the determination of monoamines, the cerebrospinal fluid samples were deproteinized with perchloric acid before centrifugation. An aliquot of the supernatant was analyzed by high pressure liquid chromatography (HPLC) with electrochemical detection. This analysis was performed as duplicate determination; the mean of the two determinations was used for further analysis and interpretation. A more detailed description of the methodology can be found in Kanitz et al. [60].

An overview of all collected variables and a short description can be found in Table 2. In the clinical as well as in the pathological examination, in theory, other variables could have been included as well, if other findings had occurred in the animals as the protocols include the thorough examinations of all organ systems. Hence, in Table 2, only variables linked to the health status are listed that were actually observed in at least one of the animals in this study. Variables were either on a continuous scale or else, categorised to be binominal (absence, presence).

**Table 2:** Overview of assessed variables on the animals

	Variable	Description	Scale	Code
Clinical examination	Rectal temperature	Body temperature measured rectally by thermometer [°C]	Continuous	1
	General behaviour: calm	Evaluation of general behaviour during general examination, any behaviour noticeably calmer than usual	Binominal	2
	General behaviour: agitated	Evaluation of general behaviour during general examination, any behaviour noticeably more agitated (may also be restless, aggressive towards observer) than usual	Binominal	3
	Coughing	Any coughing occurring during general examination	Binominal	4
	Sniffing	Any sniffing occurring during general examination	Binominal	5
	Nasal discharge	Any visible sign of nasal discharge	Binominal	6
	Snout	Evaluation of snout disc (e.g. particularly dry, sticky)	Binominal	7
	Ocular discharge	Any sign of ocular discharge	Binominal	8
	Ear veins	Noticeably pronounced ear veins	Binominal	9
	Respiratory rate	1 min count of respiratory rate by visual assessment of uplifting of flanks	Continuous	10
	Heart rate	Auscultation behind left elbow, 1 min count of heart beat	Continuous	11
	Heartbeat: intensity decreased	Decreased intensity	Binominal	12
	Heartbeat: intensity increased	Increased intensity	Binominal	13
	Abdominal breathing	Breathing is heavy and laboured, chest ring is visible with each breath	Binominal	14
	Feces	Any deviation of normal feces (e.g. consistency, colour, admixtures)	Binominal	15
	Skin condition	Any deviation in skin condition (e.g. discolouration, inflammation, abscesses)	Binominal	16
	Bristles	Any deviation (e.g. overlong, dull, density)	Binominal	17
	Breathing noise (upper lung border)	Auscultation of both sides of the lungs at superior frontal border of the lungs, any more than normal breathing sound is noted as breathing noise	Binominal	18
	Breathing noise (medium lung border)	Auscultation of both sides of the lungs at medium frontal border of the lungs, any more than normal breathing sound is noted as breathing noise	Binominal	19
	Breathing noise (lower lung border)	Auscultation of both sides of the lungs at anterior frontal border of the lungs, any more than normal breathing sound is noted as breathing noise	Binominal	20
	Conjunctivae: pale	Evaluation of colour of conjunctivae, any sign of paler colour than normal	Binominal	21
	Conjunctivae: reddened	Evaluation of colour of conjunctivae, any sign of more intense colour than normal	Binominal	22
	Episcleral vessels	Deviation in appearance (e.g. blurred, pronounced)	Binominal	23
	Tail: Skin irritation	Skin irritation (e.g. reddening, scales) of tail/tail tip	Binominal	24
	Tail: Bleeding	Blood visible on tail/tail tip	Binominal	25
	Tail: Necrosis	Signs of necrosis (e.g. constriction, dead tissue, brownish-black discolouration, dryness) on tail/tail tip	Binominal	26
	Tail: Partial loss	Tail is not in full length	Binominal	27
	Tail: Crust	Formation of crust on tail/tail tip	Binominal	28
	Tail posture	Evaluation of tail posture (curled, raised, hanging, tucked under, wagging), any other than curled or raised is evaluated as deviation	Binominal	29
	Abdominal tension	Any increase in abdominal tension assessed by palpation	Binominal	30
	Sunken flanks	Flanks appear caved-in	Binominal	31
	Prepuce	Any abnormality of prepuce (e.g. scales, abrasions, fluid, adhesion)	Binominal	32
	Body condition score	Assessed on a five point scale, 3 is normal, 1, 2 thin, 4, 5 overweight	Numerical	33
	Lnn. inguinalis superficialis	Assessed by palpation, normal size depends on size (age) of the animal, any enlargement is evaluated here	Binominal	34
	Lameness	Any visible deviation from normal stride	Binominal	35
	Joints	Any deviation in any visible joint of extremities (e.g. swelling, thickening, warmth) accessible by vision and palpation	Binominal	36
	Hernia	Any presence of hernia of any size accessible by vision and palpation (e.g. umbilical, scrotal)	Binominal	37
Pathological examination	Body weight	Weight of freshly dead whole animal	Continuous	38
	Nutritional status: thin	Nutritional status is evaluated as emaciated, moderate, normal, very well nourished, fat; emaciated and moderate is counted together for this indicator	Binominal	39
	Adrenal gland weight (right)	Adrenal glands are carefully dissected from surrounding tissue and weighed [g]	Continuous	40
	Adrenal gland weight (left)	Adrenal glands are carefully dissected from surrounding tissue and weighed [g]	Continuous	41
	Relative adrenal gland weight (right)	Adrenal gland weight / body weight	Continuous	42
	Relative adrenal gland weight (left)	Adrenal gland weight / body weight	Continuous	43
	Skin: scratches	Any scratches on any part of the skin	Binominal	44
	Skin condition	Any deviation in skin condition that are not scratches (e.g. discolouration, inflammation)	Binominal	45
	Ear: scratches	Any scratches on outer part of ear	Binominal	46
	Umbilicus	Any anomaly on the umbilicus (e.g. inflammation, fluid, thickening)	Binominal	47
	Pneumonia	Any sign of pneumonia	Binominal	48
	Lung: cyst	Fluid filled sack in lung tissue	Binominal	49
	Lung: alveolar histiocytosis	Pathohistologically visible infiltration of alveoles by histiocytes	Binominal	50
	Lung: hyperemia	Visible hyperemia	Binominal	51
	Lung: edema	Visible and palpable edema	Binominal	52
	Lung: emphysema	Visible and palpable emphysema	Binominal	53
	Pleuritis	Any sign of pleuritis	Binominal	54
	Peritoneal cavity: serous effusion	Any sign of serous effusion in peritoneal cavity	Binominal	55
	Liver: hyperemia	Visible hyperemia	Binominal	56
	Liver: hematopoiesis	Pathohistological evidence of formation of blood cells in liver tissue	Binominal	57
	Liver: lymphohistiocytic inflammation	Pathohistological evidence of inflammation process with proliferation of lymphocytes	Binominal	58
	Myocarditis	Any sign (macroscopically and pathohistologically) of myocarditis	Binominal	59
	Pericard: serous effusion	Any sign of serous effusion in pericardium	Binominal	60
	Diaphragma: lymphohistiocytic inflammation	Pathohistological evidence of inflammation, invasion of lymphohistiocytes	Binominal	61
	Kidney: lymphohistiocytic inflammation	Pathohistological evidence of inflammation, invasion of lymphohistiocytes in one or both kidneys	Binominal	62
	Kidney: cyst	Fluid filled sack in one or both kidneys	Binominal	63
	Hydronephrosis	Any macroscopically visible extension of pyelocaliceal system of one or both kidneys	Binominal	64
	Kidney: hyperemia	Any macroscopically visible sign of hyperemia in one or both kidneys	Binominal	65
	Intraocular fluidurea in aquaeus fluid of anterior chamber increase	Urea content in aquaeus fluid of the anterior chamber	Binominal	66
	Bladder: lymphohistiocytic inflammation	Pathohistological evidence of inflammation, invasion of lymphohistiocytes in bladder	Binominal	67
	Spleen: hyperplasia	Spleen is enlarged	Binominal	68
	Spleen: hyperemia	Any sign of hyperemia of spleen	Binominal	69
	Bronchus associated lymphoid tissue: hyperplasia	Enlargement of lymph nodes associated to bronchus	Binominal	70
	Colon associated lymphoid tissue: hyperplasia	Enlargement of lymph nodes associated to colon	Binominal	71
	Colon associated lymphoid tissue: crypt abscesses	Histopathologically visible crypt abscesses within the colon	Binominal	72
	Colon associated lymphoid tissue: multinucleated giant cells	Histopathologically visible infiltration of multinucleated giant cells into lymph nodes associated to colon	Binominal	73
	Pulmonal lymph nodes: hyperplasia	Enlargement of lymph nodes associated to lung	Binominal	74
	Pulmonal lymph nodes: purulent inflammation	Visible purulent inflammation of lymph nodes associated to lung	Binominal	75
	Pulmonal hemosiderosis	Visible signs of hemosiderosis in the lung tissue	Binominal	76
	Mesenteric lymph nodes: hyperplasia	Enlargement of mesenteric lymph nodes	Binominal	77
	Mesenteric lymph nodes: purulent inflammation	Visible purulent inflammation of mesenteric lymph nodes	Binominal	78
	Tonsils: hyperplasia	Enlargement of tonsils	Binominal	79
	Tonsils: crypt abscesses	Any pathohistologically visible crypt abscesses in the tonils	Binominal	80
	Tonsils: purulent inflammation	Purulent inflammation of tonsils	Binominal	81
	Lnn. gastrici: sinus histiocytosis	Pathohistologically visible hyperplasia with infiltration of histiocytes into sinus of lymph nodes associated to the stomach	Binominal	82
	Ln. sternalis: sinus histiocytosis	Pathohistologically visible hyperplasia with infiltration of histiocytes into sinus of lymph nodes associated to sternum	Binominal	83
	Tongue: inflammation	Any sign of inflammation in the tongue	Binominal	84
	Tracheitis	Any sign of inflammation in the trachea	Binominal	85
	Rhinitis	Any sign of inflammation in the nasal mucosa	Binominal	86
	Nasal discharge	Any sign of nasal discharge	Binominal	87
	Atrophic conchae	Any atrophy of conchae	Binominal	88
	Gastritis	Any sign of inflammation in the stomach	Binominal	89
	Pars proventricularis: hyperceratosis	Any sign of hyperceratosis or inflammation in the pars proventricularis	Binominal	90
	Gut: inflammation	Any visible inflammation in the gut	Binominal	91
	Gut: crypt abscesses	Pathohistologically visible crypt abscesses	Binominal	92
	Gut: intraluminal inclusion corpuscles	Pathohistologically visible intracelluar inclusion bodies	Binominal	93
	Tail: dermatitis	Any inflammation in the skin of the tail	Binominal	94
	Tail: crust	Any formation of crust on the tail	Binominal	95
	Tail: blood	Any signs of blood on the tail	Binominal	96
	Tail: necrosis	Any signs of necrosis (including pathohistological findings) on the tail	Binominal	97
	Tail: intralesional bacteria	Pathohistologically visible bacteria in present lesions	Binominal	98
	Tail: exsudation	Any sign of exsudation (including pathohistological findings) in the skin of the tail	Binominal	99
	Tail: osteomyelitis	Any signs of osteomyelitis in the tail	Binominal	100
	Ear necrosis	Any signs of necrosis on one or both ears	Binominal	101
	Skeletal musculature: inflammation	Any inflammation in all of skeletal musculature	Binominal	102
	Joints: swelling/inflammation	Any swelling and/or inflammation of any joints	Binominal	103
	Spinal cord: inflammation	Any signs of inflammation in any part of the spinal cord	Binominal	104
	Oesophagitis	Any signs of inflammation of the oesophagus	Binominal	105
	Thymus: giant cell infiltration	Multinuclear giant cell infiltration into thymus visible in pathohistological exam	Binominal	106
	N. ischiadicus: inflammation	Any sign of inflammation in one or both Nn. ischiadici	Binominal	107
	Thyreoiditis	Any sign of inflammation of the thyroid gland	Binominal	108
	Sternum: myeloid dominance	Pathohistological visible dominance of myeloid cells in sternal bone marrow	Binominal	109
	Plexus brachialis: perinerval hemorrhages	Visible bleeding in plexus brachialis	Binominal	110
	Pancreas: hemorrhage	Visible hemorrhage in pancreas	Binominal	111
	Cystitis	Any sign of inflammation of the bladder	Binominal	112
	Sheath: hyperkeratosis	Any sign of hyperkeratosis in the sheath	Binominal	113
Blood sample	Leukocytes [G/l]	Collection of standard blood parameters according to laboratory of University of Veterinary Medicine, Hanover, Foundation (TiHo)	Continuous	114
	Erythrocytes [T/l]		Continuous	115
	Haemoglobin [g/l]		Continuous	116
	Haematocrit [l/l]		Continuous	117
	Mean corpuscular volume (MCV) [fl]		Continuous	118
	Mean corpuscular haemoglobin (MCH) [pg]		Continuous	119
	Mean corpuscular haemoglobin concentration (MCHC) [g/l]		Continuous	120
	Thrombocytes [G/l]		Continuous	121
	Lymphocytes [%]		Continuous	122
	Segmented granulocytes [%]		Continuous	123
	Rod-nuclear granulocytes [%]		Continuous	124
	Metamyelocytes [%]		Continuous	125
	Eosinophilic granulocytes [%]		Continuous	126
	Basophilic granulocytes [%]		Continuous	127
	Monocytes [%]		Continuous	128
	Normoblasts [%]		Continuous	129
	Anisocytosis		Continuous	130
	Polychromacy		Binominal	131
	Poikilocytosis		Binominal	132
	Total bilirubin [µmol/l]		Continuous	133
	Conjugated bilirubin [mmol/l]		Continuous	134
	Protein [g/l]		Continuous	135
	Albumin [g/l]		Continuous	136
	Globulin: Albumin ratio		Continuous	137
	Creatinine kinase (CK) [U/l]		Continuous	138
	Aspartate-amino-transferase (ASAT) [U/l]		Continuous	139
	CK: ASAT ratio		Continuous	140
	Glutamate dehydrogenase (GLDH) [U/l]		Continuous	141
	γ glutamyl transferase (GGT)		Continuous	142
	Alkaline phosphatase (AP) [U/l]		Continuous	143
	Creatinine [µmol/l]		Continuous	144
	Urea [mmol/l]		Continuous	145
	3-hydroxybutanoic acid (3HB)		Continuous	146
	Glucose [mmol/l]		Continuous	147
	L-lactate [mmol/l]		Continuous	148
	D-lactate [mmol/l]		Continuous	149
	Calcium (Ca) [mmol/l]		Continuous	150
	Magnesium (Mg) [mmol/l]		Continuous	151
	Phosphorus (P) [mmol/l]		Continuous	152
	Sodium (Na) [mmol/l]		Continuous	153
	Potassium (K) [mmol/l]		Continuous	154
	Iron (Fe) [mmol/l]		Continuous	155
	Hemoglobin in plasma		Continuous	156
	Hemoglobin in serum		Continuous	157
	Anemia		Binominal	158
	Hypoproteinemia		Binominal	159
	Hypomagnesemia		Binominal	160
	Hyponatremia		Binominal	161
	Hypokalemia		Binominal	162
	Hyperkalemia		Binominal	163
	Hypoglycemia		Binominal	164
	Hyperglycemia		Binominal	165
	Hypocalcemia		Binominal	166
	Leukopenia		Binominal	167
	Leukocytosis		Binominal	168
	Hyperphosphatemia		Binominal	169
Cerebrospinal fluid	Dopamine [ng/ml]	Analysis of cerebrospinal fluid contents according to protocol of Research Institute for Farm Animal Biology (FBN), Dummerstorf	Continuous	170
	3,4-Dihydroxyphenylacetic acid (DOPAC) [ng/ml]		Continuous	171
	5-Hydroxyindoleacetic acid (HIAA) [ng/ml]		Continuous	172
	Homovanillic acid (HVA) [ng/ml]		Continuous	173
	Serotonin		Continuous	174

### Statistics

All statistical analyses were carried out using SAS® 9.4 [66]. As the experimental design was that whenever a biter was identified, a control animal was selected, pairwise comparisons were carried out. Each of the 174 variables was treated as independent variable and analysed separately. For each numerical variable, first the distribution of the differences of the pairs was tested by Shapiro–Wilk Test. In the case of normal distribution, paired t-Test was carried out, if normal distribution was not given, Wilcoxon signed rank test was used. For binominal variables, McNemar’s Test was carried out. The analysis of relationships between variables was again dependent on type of variable: The connections between two numerical variables were analysed by Spearman’s rank correlation coefficients. Connections between two binominal variables were evaluated via Chi^2^ Test and Cramer’s V and for analysis of connections between numerical and binominal variables, point-biserial correlation coefficients were calculated. In all cases, values were evaluated as meaningful connection if the according statistical parameter reached values of ≥ 0.40 and as strong connection if values were ≥ 0.6. The level of significance was in all cases set to *P* ≤ 0.05.

## Data Availability

The datasets used and analysed during the current study are available from the corresponding author on reasonable request.
